# Treatment protocol for pneumonia with acute kidney injury: effects of ulinastatin combined with CRRT on inflammatory factors and renal function in patients

**DOI:** 10.3389/fmed.2026.1775153

**Published:** 2026-07-24

**Authors:** Li Bai, Shuai Luo, Qiang Wang

**Affiliations:** 1 Department of Nephrology, Chongming Hospital Affiliated to Shanghai University of Medicine and Health Sciences, Shanghai, China; 2 Department of Respiratory Medicine, Chongming Hospital Affiliated to Shanghai University of Medicine and Health Sciences, Shanghai, China

**Keywords:** acute kidney injury, continuous renal replacement therapy, pneumonia, treatment protocol, ulinastatin

## Abstract

**Background:**

The optimal treatment for patients with pneumonia complicated by acute kidney injury (AKI) remains challenging. This study aims to investigate the clinical efficacy and safety of combining ulinastatin with continuous renal replacement therapy (CRRT) in these patients, thereby providing evidence for clinical treatment planning.

**Methods:**

This retrospective study included 100 patients with pneumonia complicated by AKI admitted to our hospital from January 2019 to December 2024. The assignment of patients to treatment groups was based on the regimens recorded in the electronic medical record system, as determined by the treating physicians. Patients were divided into a combination group (*n* = 49, ulinastatin plus CRRT) and a CRRT group (*n* = 51, CRRT alone). We compared post-treatment levels of inflammatory markers (interleukin [IL]-6, IL-8, C-reactive protein [CRP], tumor necrosis factor-alpha [TNF-*α*]), renal function indicators (cystatin C [CysC], blood urea nitrogen [BUN], serum creatinine [Cr]), pulmonary function indicators (maximal expiratory pressure [PEmax], maximal inspiratory pressure [Plmax], peak expiratory flow [PEF]), urine pH, and 24-h urine output between the two groups. The overall clinical response rate was also compared.

**Results:**

Post-treatment, both groups demonstrated significantly improved levels of inflammatory markers, renal function, pulmonary function, urine pH, and 24-h urine output compared to pre-treatment (*p* < 0.05). Compared to the CRRT group, the combination therapy group exhibited significantly lower post-treatment levels of IL-6 (69.43 ± 19.35 vs. 81.24 ± 18.21 pg./L, *p* < 0.05), IL-8 (148.49 ± 34.26 vs. 167.92 ± 41.44 pg./L, *p* < 0.05), CRP (81.66 ± 15.51 vs. 92.19 ± 17.23 mg/L, *p* < 0.05), TNF-*α* (103.38 ± 28.51 vs. 123.36 ± 33.22 pg./L, *p* < 0.05), BUN (7.11 ± 2.53 vs. 8.54 ± 3.81 mmol/L, *p* < 0.05), and Cr (115.45 ± 28.99 vs. 131.37 ± 35.14 μmol/L, *p* < 0.05). Post-treatment levels of CysC (8.39 ± 3.85 vs. 6.13 ± 2.72 mg/L, *p* < 0.05), PEmax (43.71 ± 5.22 vs. 35.96 ± 5.40%, *p* < 0.05), Plmax (83.34 ± 7.50 vs. 70.88 ± 6.56%, *p* < 0.05), PEF (1.57 ± 0.23 vs. 1.30 ± 0.16 L·s^−1^, p < 0.05), urine pH (6.80 ± 1.03 vs. 6.28 ± 1.12, *p* < 0.05), and 24-h urine output (1.90 ± 0.22 vs. 1.79 ± 0.21 L/24 h, p < 0.05) were significantly higher in the combination group. The overall clinical response rate in the combination group (47/49, 95.92%) was significantly higher than that in the CRRT group (42/51, 82.35%) (*p* = 0.03).

**Conclusion:**

The combination of ulinastatin and CRRT may offer additional benefits over CRRT alone in improving clinical symptoms, inflammatory markers, renal function, and pulmonary function in patients with pneumonia complicated by AKI. However, given the retrospective nature of this study, these findings should be considered preliminary and require confirmation in large-scale, prospective, randomized controlled trials.

## 1 Introduction

Pneumonia ranks among the infectious diseases with the highest incidence and mortality rates worldwide. Particularly in elderly patients, those with compromised immune function, and individuals with multiple underlying conditions, the disease often progresses rapidly and frequently escalates into severe pneumonia (1). Marzuillo P et al. (2) indicate that severe pneumonia not only causes severe respiratory failure but also serves as a major initiator of systemic inflammatory response syndrome and multiple organ dysfunction syndrome. Among the numerous affected organs, the kidneys are one of the most vulnerable targets. Acute kidney injury (AKI) is a common and severe complication of severe pneumonia, with an incidence rate as high as 20–50%. The pathogenesis of pneumonia complicated by AKI is complex, primarily involving the interplay of multiple pathophysiological processes such as inadequate renal perfusion, direct damage to renal tubular epithelial cells by pathogen toxins and inflammatory mediators, and drug-induced nephrotoxicity (3, 4). Research by Chen D et al. (5) revealed that pneumonia complicated by AKI creates a vicious cycle: AKI exacerbates internal environment disruption and fluid overload, impairing the metabolism and efficacy of antibiotics and other drugs, thereby worsening infection. Simultaneously, severe infection and inflammatory responses further damage renal function, significantly increasing treatment difficulty, prolonging hospital stays, and elevating mortality risk, posing substantial challenges in the management of critically ill patients.

Currently, clinical management of pneumonia complicated by AKI advocates combining renal replacement therapy with aggressive antimicrobial therapy, supportive treatment for the underlying disease, and protective strategies (6). Continuous renal replacement therapy (CRRT) has become a cornerstone life-support measure for treating moderate-to-severe AKI, particularly in patients with multiple organ dysfunction and hemodynamic instability. By continuously and gradually removing excess fluid and solutes from the body, CRRT enables precise regulation of volume status, effectively corrects electrolyte imbalances and metabolic acidosis, and creates a stable internal environment window for treating the underlying disease (7, 8). More importantly, CRRT’s non-selective convective and adsorption effects are believed to clear certain medium-to-large molecular inflammatory mediators from the blood, exerting a blood purification effect (9). However, numerous recent clinical studies remain controversial regarding whether CRRT significantly reduces overall mortality in patients with sepsis or severe pneumonia. Its efficiency in clearing inflammatory mediators is constrained by multiple factors including membrane material, treatment mode, and dosage, potentially insufficient to fully reverse uncontrolled systemic inflammatory responses (10–12). This indicates that CRRT alone as a physical clearance modality may be suboptimal, necessitating exploration of adjunctive approaches that regulate inflammation at its source.

Ulinastatin, a broad-spectrum serine protease inhibitor extracted from human urine, has garnered increasing attention for its anti-inflammatory and organ-protective properties. It can mitigate tissue inflammatory damage by inhibiting neutrophil activation and infiltration, stabilizing lysosomal membranes, and reducing oxygen radical release, while simultaneously modulating the activation of key inflammatory signaling pathways like nuclear factor-κB (NF-κB) to downregulate the expression and release of core pro-inflammatory factors (13, 14). This suggests that combining ulinastatin with CRRT could offer a more comprehensive strategy for controlling systemic inflammation, potentially creating a more favorable microenvironment for renal repair and improving patient outcomes. However, clinical studies on this combination therapy for pneumonia complicated by AKI remain scarce.

Therefore, this study aims to investigate the potential effects of combining ulinastatin with CRRT in patients with pneumonia complicated by AKI, with the goal of providing preliminary evidence for developing more effective treatment strategies.

## 2 Materials and methods

### 2.1 Ethical statement

This study was approved by the institutional review board and ethics committee. Given that this is a retrospective study utilizing only de-identified patient data, informed consent is not required as it poses no risk or detriment to patients. This exemption complies with regulations and ethical guidelines pertaining to retrospective research.

### 2.2 Study design

This retrospective study included 100 patients with pneumonia complicated by acute kidney injury (AKI) admitted to our hospital between January 2019 and December 2024. The assignment of patients to treatment groups was based on the regimens recorded in the electronic medical record system, as determined by the treating physicians. No randomization or predefined allocation criteria were used for this retrospective analysis. Based on these regimens, patients were divided into a combination therapy group (*n* = 49, receiving ulinastatin combined with continuous renal replacement therapy [CRRT]) and a CRRT group (*n* = 51, receiving CRRT alone).

### 2.3 Inclusion criteria

Inclusion Criteria: (1) Meets the diagnostic criteria for AKI as outlined in the Expert Consensus on Diagnosis and Classification of Acute Kidney Injury (15), confirmed by blood and urine tests; (2) Meets the pneumonia-related criteria specified in the Chinese Guidelines for Diagnosis and Treatment of Hospital-Acquired Pneumonia and Ventilator-Associated Pneumonia in Adults (16); (3) Has not recently received other antimicrobial therapy; (4) Possesses complete clinical documentation; (5) Meeting CRRT treatment indications; (6) No history of allergy to drugs used in this study.

Exclusion Criteria: (1) Concurrent malignant tumors; (2) Concurrent severe organ dysfunction (e.g., cardiac, hepatic); (3) Neurological disorders or cognitive impairment; (4) Pre-existing infection or infection incubation period at admission; (5) History of kidney transplantation; (6) Lactation or pregnancy.

### 2.4 Methods

All patients received conventional therapy, including gastrointestinal decompression, nutritional support, fluid resuscitation, correction of electrolyte imbalances, and anti-infective measures.

#### 2.4.1 CRRT group

Treatment was conducted using the continuous veno-venous hemofiltration (CVVH) mode. Vascular access was established with dual-lumen indwelling central venous catheters. The treatment frequency was set at 12–24 h per day. Replacement fluid was adjusted based on patient blood test results. Treatment employed a pre-dilution method with a pre-dilution volume of 1,500 mL/L and a post-dilution volume of 500 mL/L, with a subsequent dilution of 500 mL/L. The total replacement fluid volume ranged from 30 to 48 L, with a blood flow rate set between 150 and 300 mL/min. Ultrafiltration was adjusted based on hemodynamic indicators (2–4 L/day). Heparin was used for anticoagulation in the blood circuit. Patients with mild or significant bleeding tendencies did not receive anticoagulation therapy, while those without bleeding tendencies were administered low-dose heparin anticoagulation.

#### 2.4.2 Combined group

In addition to the CRRT regimen, patients in this group received ulinastatin (Manufacturer: Guangdong Tianpu Biochemical Pharmaceutical Co., Ltd., Approval Number: National Drug Approval No. H19990133). A solution of 200,000 units of ulinastatin in 0.9% sodium chloride was prepared and administered intravenously at 20 mL per dose, three times daily. The CRRT treatment protocol was identical to that of the CRRT group described above. All patients underwent one week of treatment, with clinically relevant indicators collected before and after treatment.

### 2.5 General data collection

General patient data were retrospectively collected via the electronic medical record system, primarily including age, gender (male/female), body mass index, hypertension [meeting diagnostic criteria per The Japanese Society of Hypertension Guidelines for Self-monitoring of Blood Pressure at Home (Second Edition) (17)] (present/absent), diabetes [meeting diagnostic criteria in Application of the Chinese Expert Consensus on Diabetes Classification in clinical practice (18)] (present/absent), hyperlipidemia [meeting diagnostic criteria in Report of the Japan Atherosclerosis Society (JAS) Guideline for Diagnosis and Treatment of Hyperlipidemia in Japanese adults (19) present/absent], and time from symptom onset to admission.

### 2.6 Inflammatory markers

Includes interleukin (IL)-6, IL-8, C-reactive protein (CRP), and tumor necrosis factor-*α* (TNF-α). Collect 5 mL of patient venous blood in the morning on an empty stomach. Centrifuge at 3000 rpm for 10 min using a centrifuge (Model: LL900, Manufacturer: Luoyang Hongshi Machinery Equipment Co., Ltd.), at 3000 rpm for 10 min to separate serum. Enzyme-linked immunosorbent assay (ELISA) was employed for detection, with procedures strictly adhering to kit instructions.

### 2.7 Renal function markers

Included cystatin C (CysC), blood urea nitrogen (BUN), and serum creatinine (Cr). Serum samples were analyzed using a fully automated biochemical analyzer (Hitachi, Model 7,650).

### 2.8 Pulmonary function parameters

Included peak expiratory pressure (PEP), peak inspiratory pressure (PIP), and peak expiratory flow rate (PEF). Measurements were performed using an MSA99 spirometer (Beijing Maibang Biotechnology Co., Ltd.).

### 2.9 Urinalysis

Includes urine pH (measured using potentiometry or dry chemistry test strips; urine samples must be brought to room temperature and thoroughly mixed prior to testing; if sediment is present, centrifuge the sample and measure the supernatant; Instruments must be calibrated daily with standard buffers (pH 4.01, 7.00) to ensure accuracy). 24-h urine volume (Begin timing after emptying the bladder in the morning and collect all urine produced over the subsequent 24 h. Store in a clean container treated with a dedicated preservative. Record the total volume, mix thoroughly, and submit an appropriate sample for testing).

### 2.10 Outcome status

Patient outcomes at the end of the one-week treatment period were categorized as cure, improvement, or death. The criteria for these outcomes were: Cure: complete resolution of symptoms and signs, normalization of blood counts, and resolution of inflammatory opacities on chest X-ray. Improvement: significant alleviation of symptoms and signs, near-normalization of blood counts, and near-resolution of inflammatory opacities on chest X-ray. The overall clinical response rate was defined as the sum of cured and improved cases.

### 2.11 Statistical methods

Statistical analyses were performed using SPSS version 25.0 (IBM Corp., Armonk, NY, USA). Categorical data are presented as frequencies (n, %) and were compared between groups using the chi-square (χ^2^) test. The normality of continuous data was assessed using the Shapiro–Wilk test. Continuous data with a normal distribution are expressed as mean ± standard deviation (x̅ ± s) and were compared using the independent samples t-test for between-group comparisons and the paired t-test for within-group comparisons. Continuous data not following a normal distribution are expressed as median and interquartile range [M (P25, P75)] and were analyzed using the Mann–Whitney U test for between-group comparisons. No *a priori* sample size calculation or power analysis was performed, as this study was retrospective and exploratory in nature, including all eligible patients from the specified period. No multivariable adjustments or propensity score matching were performed due to the study’s retrospective design and the limited sample size, which increases the risk of residual confounding. A two-tailed *p* value < 0.05 was considered statistically significant.

## 3 Results

### 3.1 Comparison of general patient characteristics between the combined group and CRRT group

There were no statistically significant differences between the combined group and CRRT group in baseline characteristics including age, gender, body mass index, hypertension, diabetes, hyperlipidemia, and time from onset to admission (*p* > 0.05). See Table 1.

**Table 1:** Comparison of general data of patients in the combined group and the CRRT group.

Indicator		Joint group (*n* = 49)	CRRT group (*n* = 51)	*x^2^/t*	*p*
Age (years, *x_*±s)		63.37 ± 7.12	62.97 ± 7.34	0.276	0.783
Gender (*n*, %)	Male	28 (57.14)	30 (58.82)	0.029	0.865
	Female	21 (42.86)	21 (41.18)		
Body Mass Index (kg/m^2^, *x_*±s)		23.95 ± 1.88	24.01 ± 1.74	0.166	0.869
Hypertension (*n*, %)	Have	6 (12.24)	4 (7.84)	0.538	0.463
	No	43 (87.76)	47 (92.16)		
Diabetes (*n*, %)	Have	3 (6.12)	5 (9.80)	0.460	0.498
	No	46 (93.88)	46 (90.20)		
Hyperlipidemia (*n*, %)	Have	4 (8.16)	2 (3.92)	0.200	0.655
	No	45 (91.84)	49 (96.08)		
The time from the onset of the disease to admission (h, *x_*±s)		10.05 ± 3.19	10.11 ± 2.88	0.099	0.921

### 3.2 Comparison of inflammatory markers

As presented in Table 2, baseline levels of IL-6, IL-8, CRP, and TNF-*α* were comparable between the two groups before treatment (p > 0.05).

**Table 2:** Comparison of inflammatory indicators before treatment between the combined group and the CRRT group (*x_* ± s).

Indicator	Joint group (*n* = 49)	CRRT group (*n* = 51)	*t*	*p*
IL-6 (pg/L)	214.88 ± 55.67	216.72 ± 54.36	0.167	0.868
IL-8 (pg/L)	602.26 ± 110.62	599.19 ± 101.29	0.145	0.885
CRP (mg/L)	118.62 ± 32.39	117.56 ± 29.59	0.171	0.865
TNF-α (pg/L)	301.12 ± 55.59	299.87 ± 51.43	0.117	0.907

**Figure 1:**
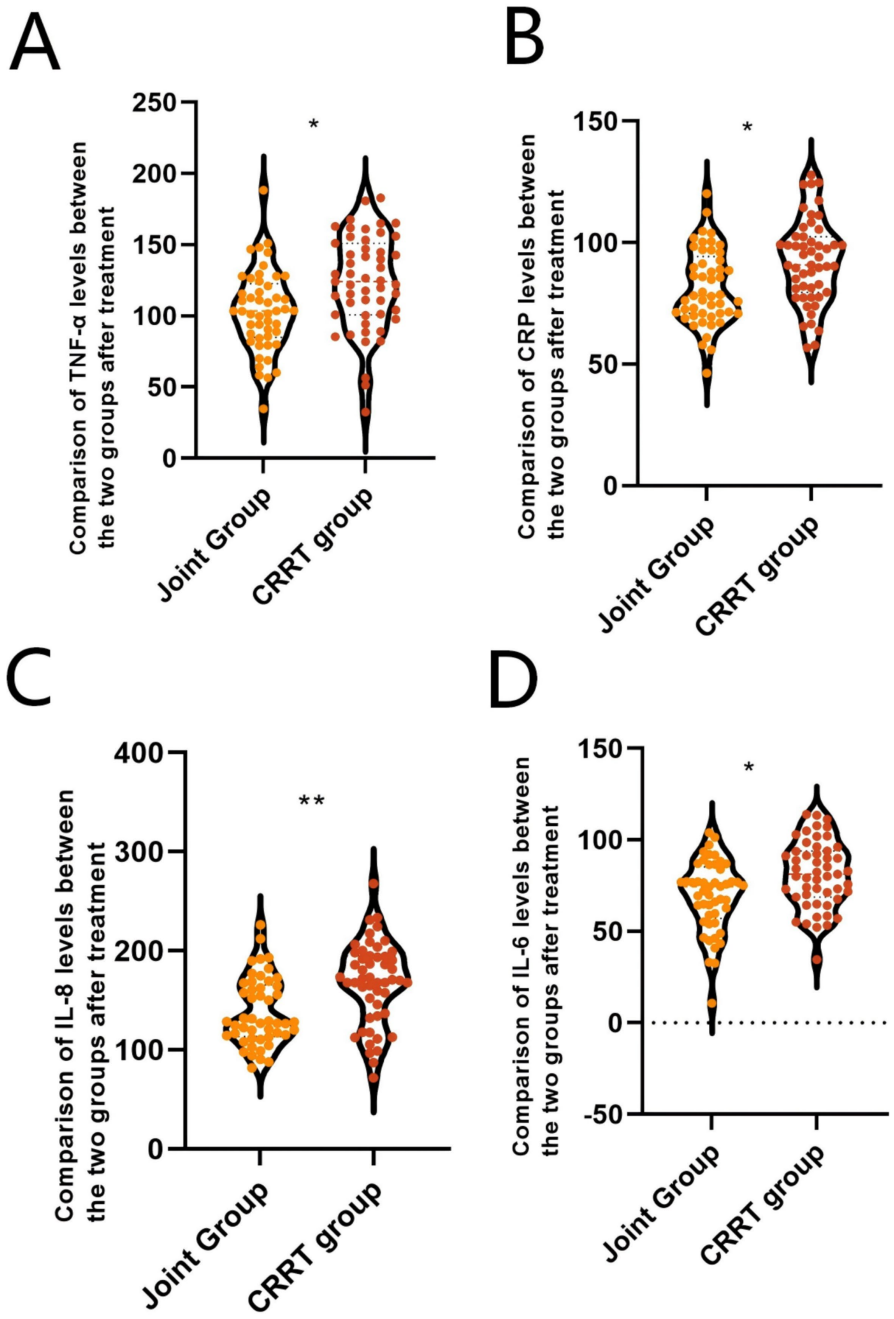
Comparison of inflammatory indicators after treatment between the combined group and the CRRT group. The violin plots illustrate the full distribution of the data, with the white dot representing the median, the thick black bar the interquartile range, and the thin black line the rest of the distribution, excluding outliers. ^*^p < 0.05, ^**^p < 0.001. **(A)** Comparison of TNF-α levels between the two groups after treatment; **(B)** Comparison of CRP levels between the two groups after treatment; **(C)** Comparison of IL-8 levels between the two groups after treatment; **(D)** Comparison of IL-6 levels between the two groups after treatment.

Following treatment, both groups showed significant improvements in all inflammatory markers compared to their respective baseline levels (*p* < 0.05). Importantly, as illustrated in Figure 1, the combination group exhibited significantly lower post-treatment levels of IL-6 (69.43 ± 19.35 vs. 81.24 ± 18.21 pg./L, *p* < 0.05), IL-8 (148.49 ± 34.26 vs. 167.92 ± 41.44 pg./L, p < 0.05), CRP (81.66 ± 15.51 vs. 92.19 ± 17.23 mg/L, p < 0.05), and TNF-*α* (103.38 ± 28.51 vs. 123.36 ± 33.22 pg./L, p < 0.05) compared to the CRRT group. This suggests that the addition of ulinastatin to CRRT is associated with a greater attenuation of the systemic inflammatory response.

### 3.3 Comparison of renal function indicators

Baseline renal function, as measured by CysC, BUN, and Cr, was similar between the two groups (*p* > 0.05, Table 3).

**Table 3:** Comparison of renal function indicators before treatment in the combined group and the CRRT group (*x_* ± s).

Indicator	Joint group (*n* = 49)	CRRT group (*n* = 51)	*t*	*p*
CysC (mg/L)	3.01 ± 0.54	2.95 ± 0.66	0.496	0.621
BUN (mmol/L)	69.01 ± 19.52	68.32 ± 18.25	0.183	0.855
Cr (μmol/L)	801.35 ± 142.97	799.35 ± 141.34	0.070	0.944

**Figure 2:**
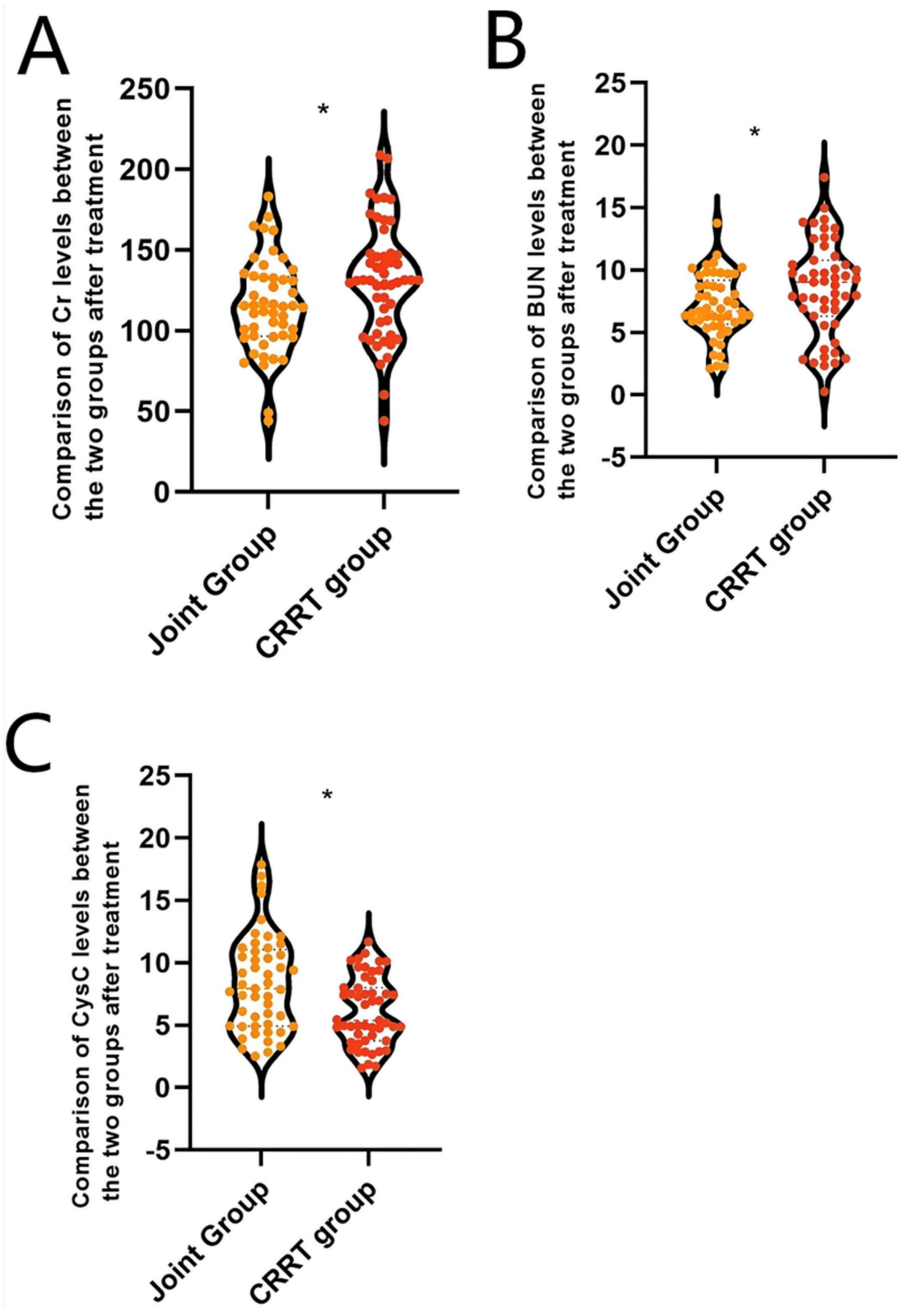
Comparison of renal function indicators between the combined group and the CRRT group after treatment. The corrected post-treatment CysC value for the combination group is 3.98 ± 1.25 mg/L and for the CRRT group is 5.21 ± 1.68 mg/L. ^*^p < 0.05, ^**^p < 0.001. **(A)** Comparison of Cr levels between the two groups after treatment; **(B)** Comparison of BuN levels between the two groups after treatment; **(C)** Comparison of CysC levels between the two groups after treatment.

Post-treatment, significant improvements in all renal function parameters were observed within each group compared to baseline (*p* < 0.05). As shown in Figure 2, the combination group demonstrated significantly lower post-treatment levels of BUN (7.11 ± 2.53 vs. 8.54 ± 3.81 mmol/L, *p* < 0.05) and Cr (115.45 ± 28.99 vs. 131.37 ± 35.14 μmol/L, p < 0.05) compared to the CRRT group. Conversely, post-treatment CysC levels were significantly lower in the combination group than in the CRRT group (3.98 ± 1.25 vs. 5.21 ± 1.68 mg/L, p < 0.05). This finding, consistent with the improvements in BUN and Cr, suggests that the combination of ulinastatin and CRRT is associated with enhanced recovery of renal function.

### 3.4 Comparison of pulmonary function indicators between the combined group and CRRT group

PEmax, Plmax, and PEF are key indicators for assessing respiratory muscle strength and airway patency in patients with pneumonia complicated by acute kidney injury. Before treatment, comparisons of PEmax, Plmax, and PEF levels between the combined group and CRRT group showed no statistically significant differences (*p* > 0.05), as shown in Table 4.

**Table 4:** Comparison of pulmonary function indicators before treatment in the combined group and the CRRT group (*x_* ± s).

Indicator	Joint group (*n* = 49)	CRRT group (*n* = 51)	*t*	*p*
PEmax (%)	30.61 ± 3.98	30.21 ± 3.83	0.512	0.610
Plmax (%)	60.12 ± 5.61	60.09 ± 5.97	0.026	0.979
PEF (L·s^−1^)	1.09 ± 0.15	1.07 ± 0.19	0.583	0.561

**Figure 3:**
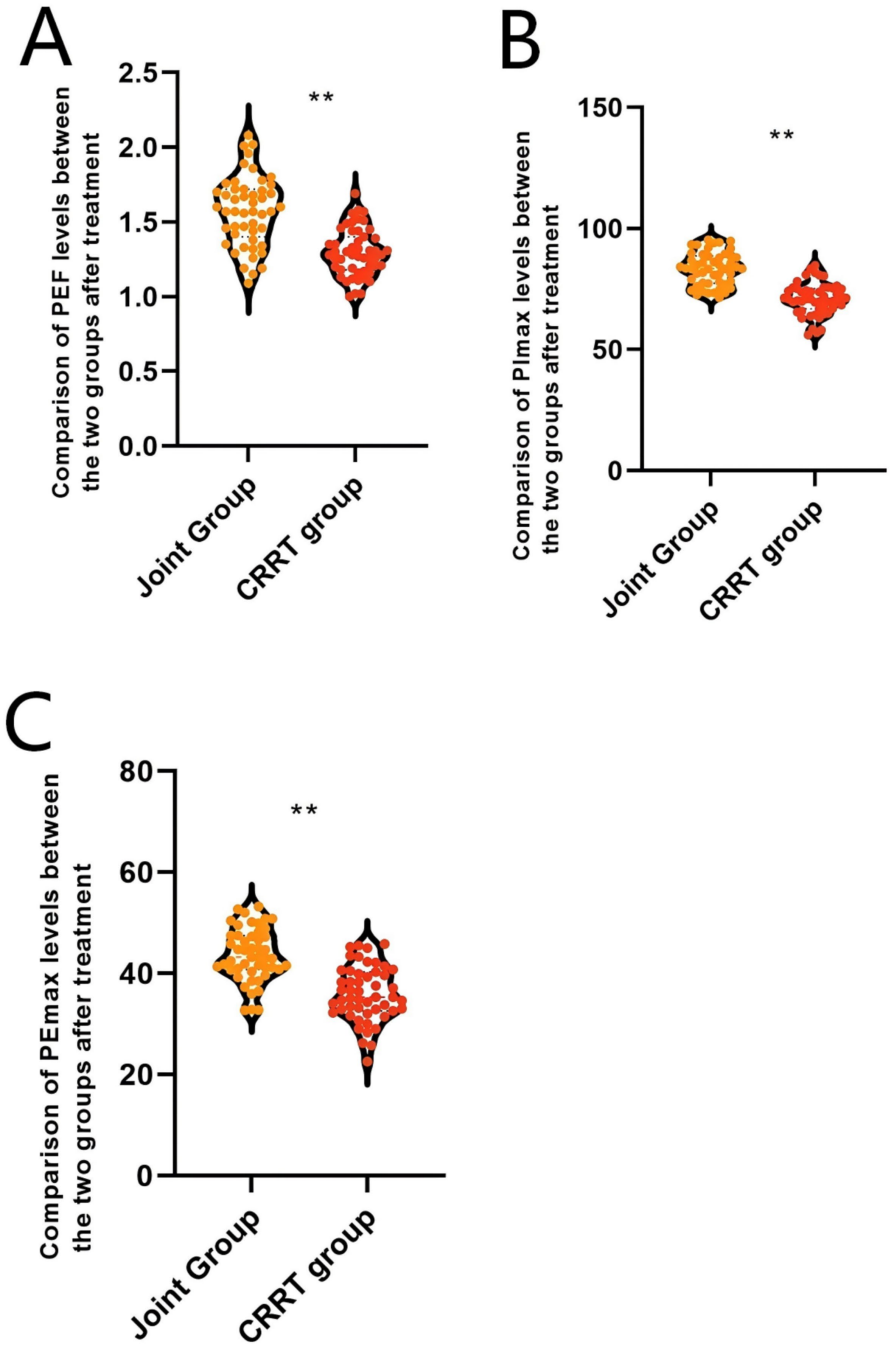
Comparison of pulmonary function indicators between the combined group and the CRRT group after treatment. ^*^p < 0.05, ^**^p < 0.001. **(A)** Comparison of PEF levels between the two groups after treatment; **(B)** Comparison of Plmax levels between the two groups after treatment; **(C)** Comparison of PEmax levels between the two groups after treatment.

Following treatment, combined PEmax ((43.71 ± 5.22 vs. 35.96 ± 5.40) %), Plmax ((83.34 ± 7.50 vs. 70.88 ± 6.56)%), PEF ((1.57 ± 0.23 vs. 1.30 ± 0.16) L·s^−1^) were significantly higher than those in the CRRT group (*p* < 0.05), indicating that the combination of ulinastatin and CRRT effectively improves pulmonary function in patients, as shown in Figure 3.

### 3.5 Comparison of urinary parameters between the combined group and CRRT group

24-h urine output directly reflects renal perfusion and recovery from injury, with increased output indicating improved renal function. Urine pH indirectly reflects tubular acid secretion and acid–base regulation capacity, and its changes suggest tubular function recovery and internal environment stability. Both parameters provide objective evidence for assessing treatment response and prognosis. Prior to treatment, comparisons of urinary pH and 24-h urine output between the combined therapy group and CRRT group showed no statistically significant differences (*p* > 0.05), as shown in Table 5.

**Table 5:** Comparison of urine conditions of patients in the combined group and the CRRT group before treatment (*x_* ± s).

Indicator	Joint group (*n* = 49)	CRRT group (*n* = 51)	*t*	*p*
24-h urine output (L/24 h)	1.21 ± 0.13	1.20 ± 0.10	0.432	0.667
Urine pH	2.40 ± 0.12	2.41 ± 0.11	0.435	0.665

**Figure 4:**
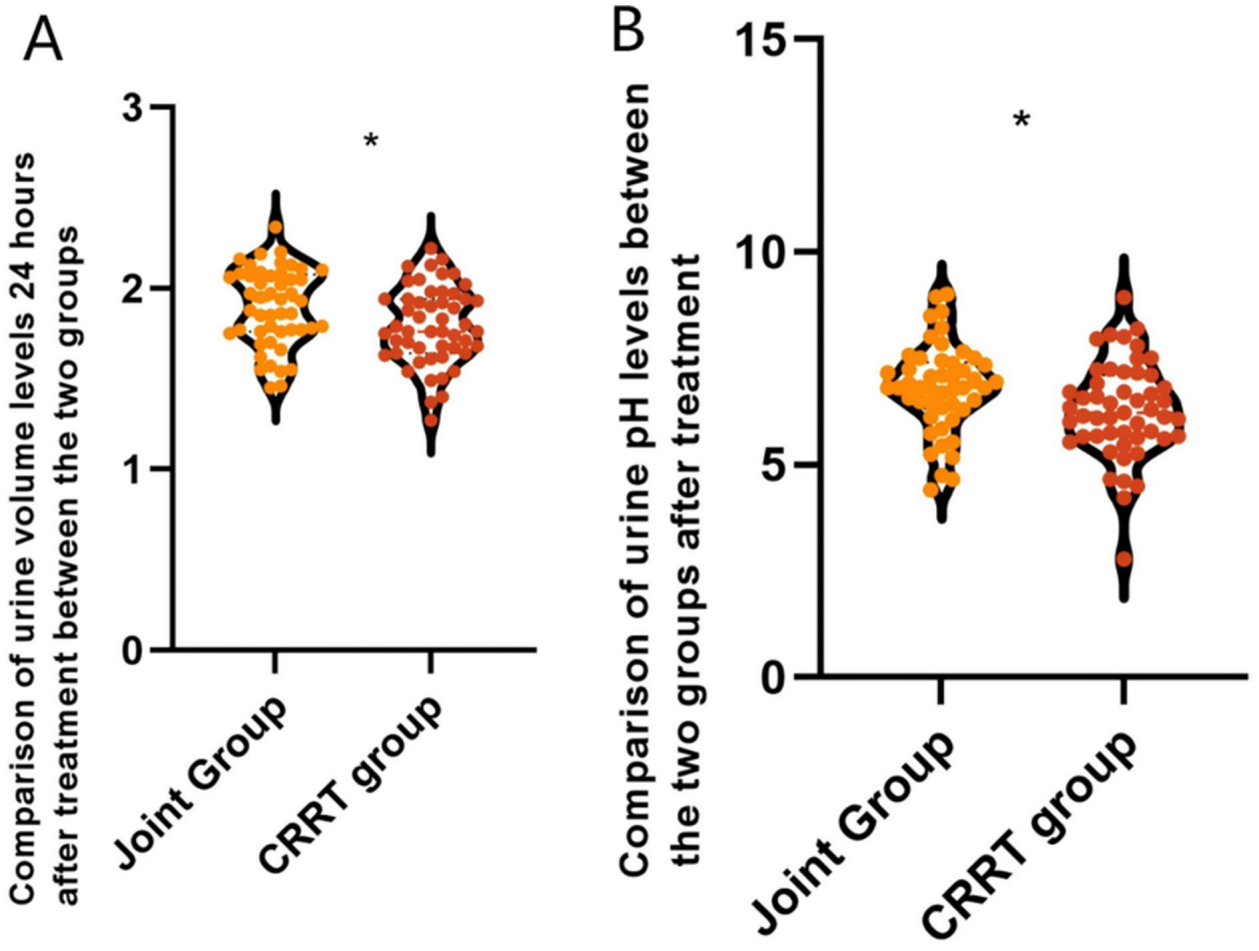
Comparison of urine conditions after treatment between the combined group and the CRRT group. ^*^p < 0.05, ^**^p < 0.001. **(A)** Comparison of urine volume levels 24 hours after treatment between the two groups; **(B)** Comparison of urine pH levels between the two groups after treatment.

Following treatment, the combined group exhibited significantly higher 24-h urine output ((1.90 ± 0.22 vs. 1.79 ± 0.21) L/24 h) and urine pH (6.80 ± 1.03 vs. 6.28 ± 1.12) compared to the CRRT group (*p* < 0.05). This indicates that the combination of ulinastatin and CRRT effectively improves urinary parameters in patients, as shown in Figure 4.

### 3.6 Comparison of outcomes between the combined group and CRRT group

Cure, improvement, and death represent disease outcomes. Higher rates of cure and improvement coupled with lower mortality indicate superior clinical treatment efficacy. The combined group demonstrated a higher overall clinical response rate (47.95% vs. 42.82%) than the CRRT group, with a statistically significant difference (*p* < 0.05). This suggests that the combination of ulinastatin and CRRT effectively promotes clinical outcomes, as shown in Table 6.

**Table 6:** Comparison of the prognosis of patients in the combined group and the CRRT group (*n*, %).

Indicator	Joint group (*n* = 49)	CRRT group (*n* = 51)	*t*	*p*
Cure	15 (30.61)	14 (27.45)	—	—
Improvement	32 (65.31)	28 (54.90)	—	—
Death	2 (4.08)	9 (17.65)	—	—
Total effective rate	47 (95.92)	42 (82.35)	4.697	0.030

## 4 Discussion

The pathophysiological mechanisms in patients with pneumonia complicated by acute kidney injury involve complex interactions between systemic inflammatory responses and multi-organ dysfunction. Among these, IL-6 serves as an early warning marker. It activates the JAK–STAT pathway to promote hepatic synthesis of acute-phase proteins such as CRP, while simultaneously inducing vascular endothelial cells to express adhesion molecules, thereby exacerbating pulmonary vascular permeability and damaging the glomerular filtration barrier. IL-8, acting as a chemokine, recruits neutrophils to lung tissue where they release reactive oxygen species, disrupting the alveolar-capillary barrier and triggering tubulointerstitial inflammation. TNF-*α* amplifies the inflammatory cascade via the NF-κB pathway, promoting tubular epithelial cell apoptosis and directly worsening renal function. CRP, as a non-specific inflammatory marker, exhibits persistently elevated levels indicating disease progression and may exacerbate glomerular injury by activating the complement system (20, 21). Abnormally elevated inflammatory factors inhibit sodium channel activity in renal tubular epithelial cells, reducing renal blood flow and subsequently activating the renin-angiotensin system. This leads to early impairment of glomerular filtration function and disruption of renal compensatory mechanisms, negatively affecting urine pH and 24-h urine output. Simultaneously, by inhibiting mitochondrial energy metabolism in muscle cells, it causes decreased diaphragm contractility and enhanced catabolism of respiratory muscle proteins, mediating respiratory muscle dysfunction. This inhibition of mitochondrial energy metabolism in muscle cells, leading to reduced diaphragm contractility and increased respiratory muscle protein catabolism, forms the pathological basis for the vicious cycle of inflammation-organ dysfunction (22). Therefore, implementing scientifically effective treatments holds significant importance for patients.

Based on the data from this study, post-treatment levels of IL-6, IL-8, CRP, TNF-*α*, BUN, Cr, CysC, PEmax, Plmax, PEF, urine pH, and 24-h urine output in the CRRT group were significantly superior to pre-treatment levels (*p* < 0.05). This indicates that CRRT treatment effectively ameliorates inflammatory responses while promoting renal and pulmonary function. This improvement stems from CRRT’s role as a novel blood purification method that significantly enhances patients’ immune function. CRRT is a therapy that replaces renal function through slow, continuous blood purification. Its mechanism of action mimics glomerular filtration by performing hemofiltration based on the filtration principle of the glomerular unit. During the extracorporeal simulation process, large volumes of fluid similar to plasma and extracellular fluid components are replenished to substitute for tubular function. Primarily used in the treatment of critically ill patients, CRRT stabilizes hemodynamics, precisely control the internal environment, continuously eliminate toxins, and improve renal function (23, 24). CRRT treatment rapidly removes metabolic byproducts, endotoxins, various inflammatory mediators, and immune activation factors through diffusion, convection, and adsorption. It eliminates circulating cytokines while reducing their tissue concentrations, thereby halting cytokine cascade reactions. This process is unaffected by ultrafiltration volume and other factors, mitigating inflammatory responses and delaying multi-organ dysfunction caused by these mediators. Consequently, it reduces tissue damage and restores internal environmental homeostasis (25). Furthermore, CRRT removes excess fluid and solutes via convective diffusion through semipermeable membranes, corrects electrolyte imbalances, and eliminates inflammatory mediators. This mitigates systemic inflammatory damage to lung tissue, thereby improving oxygenation and indirectly alleviating pulmonary edema and respiratory burden. Furthermore, CRRT replaces the filtration function of impaired kidneys, promoting the excretion of metabolic waste products such as creatinine and blood urea nitrogen. This reduces the risk of tubular obstruction, restores urine production capacity, and consequently improves 24-h urine output and urine pH levels.

However, data from this study indicate that post-treatment levels of IL-6, IL-8, CRP, TNF-*α*, BUN, and Cr were lower in the combined therapy group than in the CRRT group (*p* < 0.05). Post-treatment levels of CysC, PEmax, Plmax, PEF, urine pH, and 24-h urine output were higher in the combined therapy group than in the CRRT group (p < 0.05). The overall clinical response rate in the combination group (47 [95.92]) was higher than that in the CRRT group (42 [82.35]), with a statistically significant difference (p < 0.05). This indicates that adding usultadine to CRRT treatment yields superior clinical efficacy. Ursodeoxycholic acid is an endogenous anti-inflammatory substance associated with innate immunity. It exerts multiple protective effects during SPS treatment, including organ protection, anti-inflammation, and enzyme inhibition. Containing pro-α1-antitrypsin and inter-α1-antitrypsin, it binds to serine proteases to exert anti-toxic protease effects, protecting cells and tissues, alleviating systemic excessive stress-induced inflammatory responses, and mitigating tissue and organ damage caused by inflammation (26, 27). Additionally, usultadine exhibits specificity toward multiple hydrolases, maximally reducing tissue injury caused by hydrolase activation. It effectively suppresses superoxide production while scavenging excess superoxide within the body, eliminating free radicals, enhancing immune function, and protecting renal function, resulting in superior clinical efficacy (28). Therefore, the combined use of ulinastatin and CRRT enhances the clearance of toxins and inflammatory mediators, promotes internal environment stability, and exerts a synergistic effect to improve patient outcomes. Preclinical studies have demonstrated that ulinastatin mitigates gentamicin-induced renal tubular epithelial cell damage by alleviating oxidative stress, antagonizing inflammatory responses, and suppressing apoptosis (29). Previous studies indicate that as a broad-spectrum serine protease inhibitor, ulinastatin exhibits potent anti-inflammatory and cytoprotective effects. It is thought to regulate key intracellular signaling pathways, such as nuclear factor-κB (NF-κB) and phosphoinositide 3-kinase/protein kinase B/nuclear factor erythroid 2-related factor 2 (PI3K/Akt/Nrf2), which play pivotal roles in inflammation, oxidative stress, and apoptosis (30, 31). Through these mechanisms, ulinastatin may reduce the secretion of inflammatory factors, stabilize lysosomal membranes, and mitigate renal injury.

This study has several limitations that must be acknowledged. First, its retrospective, single-center design with a relatively small sample size (n = 100) limits the generalizability of our findings and makes them susceptible to selection bias. The allocation to treatment groups was based on physician discretion rather than randomization, and we cannot exclude the possibility that unmeasured confounders, such as differences in baseline disease severity (e.g., SOFA or APACHE II scores, AKI stage, pneumonia severity), influenced the outcomes. Second, the absence of multivariable or propensity score-adjusted analyses means that the reported treatment effects may be confounded. Third, the short follow-up period (one week) precludes any assessment of long-term outcomes, including sustained renal recovery, need for chronic dialysis, or long-term mortality. Given these significant limitations, our findings should be considered preliminary and hypothesis-generating. They underscore the need for well-designed, large-scale, multicenter, prospective randomized controlled trials with extended follow-up periods to definitively evaluate the efficacy and safety of this combination therapy and to provide a robust evidence base for clinical decision-making.

## 5 Conclusion

In summary, the combination of ulinastatin and CRRT demonstrates significant clinical value in patients with pneumonia complicated by AKI. This approach effectively improves urinary output and alleviates inflammatory responses while enhancing renal and pulmonary function, thereby promoting favorable clinical outcomes.

## Data Availability

The original contributions presented in the study are included in the article/supplementary material, further inquiries can be directed to the corresponding author.
